# Identification and Characterization of Alternative Promoters, Transcripts and Protein Isoforms of Zebrafish R2 Gene

**DOI:** 10.1371/journal.pone.0024089

**Published:** 2011-08-24

**Authors:** Hanqiao Shang, Qing Li, Guohui Feng, Zongbin Cui

**Affiliations:** 1 The Key Laboratory of Aquatic Biodiversity and Conservation of Chinese Academy of Sciences, Institute of Hydrobiology, Chinese Academy of Sciences, Wuhan, Hubei, People's Republic of China; 2 Graduate University of the Chinese Academy of Sciences, Beijing, People's Republic of China; Tulane University Health Sciences Center, United States of America

## Abstract

Ribonucleotide reductase (RNR) is the rate-limiting enzyme in the de novo synthesis of deoxyribonucleoside triphosphates. Expression of RNR subunits is closely associated with DNA replication and repair. Mammalian RNR M2 subunit (R2) functions exclusively in DNA replication of normal cells due to its S phase-specific expression and late mitotic degradation. Herein, we demonstrate the control of R2 expression through alternative promoters, splicing and polyadenylation sites in zebrafish. Three functional R2 promoters were identified to generate six transcript variants with distinct 5′ termini. The proximal promoter contains a conserved E2F binding site and two CCAAT boxes, which are crucial for the transcription of R2 gene during cell cycle. Activity of the distal promoter can be induced by DNA damage to generate four transcript variants through alternative splicing. In addition, two novel splice variants were found to encode distinct N-truncated R2 isoforms containing residues for enzymatic activity but no KEN box essential for its proteolysis. These two N-truncated R2 isoforms remained in the cytoplasm and were able to interact with RNR M1 subunit (R1). Thus, our results suggest that multilayered mechanisms control the differential expression and function of zebrafish R2 gene during cell cycle and under genotoxic stress.

## Introduction

Ribonucleotide reductase (RNR) is the rate-limiting enzyme to catalyze the de novo synthesis of deoxyribonucleoside triphosphates (dNTPs) by reducing four ribonucleoside diphosphates (NDPs) to their corresponding deoxyribonucleoside diphosphates (dNDPs). These dNDPs are then phosphorylated to their 5′-triphosphate forms. Thus, RNR provides the fundamental nucleotide building blocks for DNA synthesis and repair in all living organisms. RNRs are divided into three classes according to their mechanisms for radical generation. Nearly all eukaryotes have a class I RNR, which is a heterotetramer composed of two large and two small subunits. Both large and small subunits are required for the enzymatic activity. The large subunit contains one catalytic active site and two allosteric sites for allosteric effectors. The small subunit contributes a binuclear iron center and a tyrosyl free radical that are essential for catalysis [1]. It has been shown that unbalanced dNTPs supply can lead to genetic abnormalities and cell death [2]. Therefore, functions and expression regulation of RNR subunits from yeast to mammals have attracted extensive attention due to their critical roles in DNA synthesis and repair.

Budding yeast (*S. cerevisiae*) has two large subunits (R1 and R3) and two small subunits (R2 and R4). R1 is essential for mitotic viability and its transcription is regulated in a cell cycle-specific manner and can be induced by DNA damage [3]. R3 transcript is nearly absent during normal growth, but highly induced after DNA damage; this transcript plays a significant role in genotoxic stress [4]. R2 and R4 can be regulated in a cell cycle-specific manner and induced by DNA damage. R2 and R4 are essential for mitotic growth [5]. R4 lacks several conserved residues required for enzymic activity, but it works together with R2 to form a functional heterodimer [6]. Inhibitory proteins competing with R2 and R4 for the large subunit and the nucleus-to-cytoplasm redistribution of small subunits can also regulate RNR activity [7]. In addition, fission yeast (*S. pombe*) contains one large and one small RNR subunit, cdc22 and suc22, respectively [8]. Inhibitory regulation of the large subunit, redistribution of small subunit and a unique posttranscriptional control are also shown to regulate RNR activity in fission yeast [9], [10].

In higher plants, tobacco contains at least two R1 subunits and one R2, all of which are transcribed in a cell cycle-specific manner and mediated by E2F sites [11], [12], [13]. E2F sites also mediate the induced transcription of R1a gene and subcellular relocalization of R1a protein upon UV-C irradiation [14]. Arabidopsis has one R1 and three small subunits: AtTSO2, AtR2A and AtR2B. These small subunits display a degree of functional redundancy, but AtTSO2 normally plays a more predominant role than AtR2A and AtR2B. AtR2B is truncated in the N-terminal region and some residues involved in catalytic activity are missing and modified [15]. Transcription of AtTSO2 and AtR2A are S phase-specific and genes encoding three small subunits are differentially expressed in response to genotoxins [16].

Mammals contain one large subunit R1 and two small subunits: R2 and the newly identified p53R2. Levels of R1 are nearly constant throughout the cell cycle and in excess relative to that of R2 [17]. The enzymatic activity of RNR is therefore controlled by the level of R2. R2 is specifically transcribed during S phase through cell cycle-associated factors [18], [19], and degraded in late mitosis by a Cdh1-APC-mediated proteolysis via a KEN box in its N terminal [20]. Thus, it is suggested that R2 mainly supplies dNTPs for the nuclear DNA replication during S-phase [18]. Although expression of R2 gene is not induced by DNA damage in normal cells [21], it is upregulated in some cancer cells to supply dNTPs for DNA damage repair due to impaired p53-dependent induction of p53R2 [22]. p53R2 is a transcriptional target in ATM/CHK2 pathways and is markedly induced by p53 after DNA damage [23], [24]. p53R2 contains no KEN box and is stabilized after DNA damage through an ATM dependent mechanism [25]. However, p53R2 is constitutively expressed at a low level throughout the cell cycle under normal conditions [26]. In addition to its role in supplying dNTPs for DNA damage repair, p53R2 plays crucial roles in supplying cells outside of the S phase with dNTPs for “everyday” DNA repair as a result of oxidative damage and depurination, and for mitochondrial DNA replication [27], [28].

The zebrafish (*Danio rerio*) has been widely accepted as an ideal model for genetics, developmental biology, mechanisms of human diseases and drug discovery [29]. Molecular features of zebrafish R1 and R2 were previously described [30], but expression and functions of RNR subunit genes in zebrafish remain largely unknown. We have recently revealed the expression and functions of zebrafish p53R2 in response to DNA damage [31]. In this study, we aimed to uncover molecular mechanism(s) underlying the expression of zebrafish R2 gene during the cell cycle and in response to DNA damage.

## Results

### In silico analysis of the 5′- and 3′- flanking regions of zebrafish R2 gene

**Figure 1:**
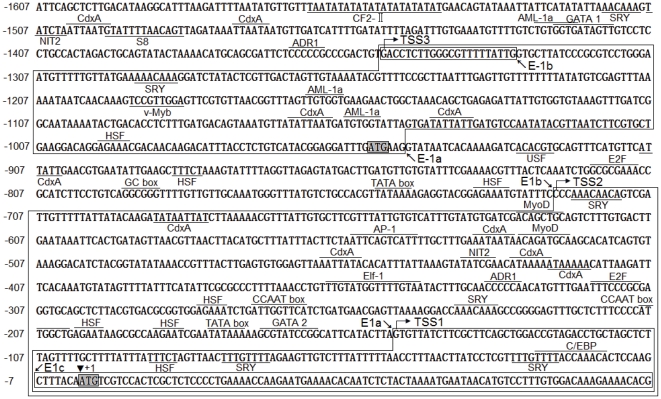
5′-flanking region of zebrafish R2 locus. Nucleotides are numbered with the first nucleotide of the proximal ATG designated as +1 (indicated by solid triangle). Two putative translational initiation sites (ATGs) are shadowed. Potential binding sites for a variety of transcription factors are underlined or overlined. Three alternative transcriptional start sites (TSS1, TSS2 and TSS3) are indicated by rightwards arrows. Exons (E-1a, E-1b, E1a, E1b and E1c) are boxed.

To address the transcriptional regulation of R2 gene, we first performed a promoter prediction algorithm for the 5′-regulatory sequence of R2 gene in zebrafish. Three putative transcriptional start sites were found and two of them (TSS1 and TSS2) are shown in Figure 1. Another TSS was eventually proved to be a false-positive predication by our RT-PCR analysis of its transcriptional products and promoter activity detection (data not shown). In addition, *in silico* cloning based on ESTs in the Genbank database revealed the third transcriptional start site (TSS3, Figure 1) for zebrafish R2 gene.

Numerous potential transcriptional factor binding sites including TATA box, CCAAT box, E2F, SRY, GC box, HSF, GATA2, AP-1, CdxA, MyoD, Elf-1, NIT2, USF, ADR1, GATA1, S8, CF2-II, cap, NIT2, Sox-5, Dfd, Oct-1, AML-1a and BR-CZ, were found in three potential promoter regions, designated P1, P2 and P3. P1 and P2 contain a typical TATA-box sequence, but no TATA-box consensus sequence was found in the core region of P3.

Moreover, two functional polyadenylation sites (pAS1 and pAS2) in the 3′-most exon of R2 gene were found through bioinformatic analysis of existing cDNA/ESTs (Figure S1A) and functional elements around pAS1 and pAS2 are highly conserved as shown in previous studies [32].

Thus, the existence of alternative potential promoters and polyadenylation sites suggests multiple transcript variants for R2 gene in zebrafish.

### Identification of R2 transcript variants in zebrafish

**Figure 2:**
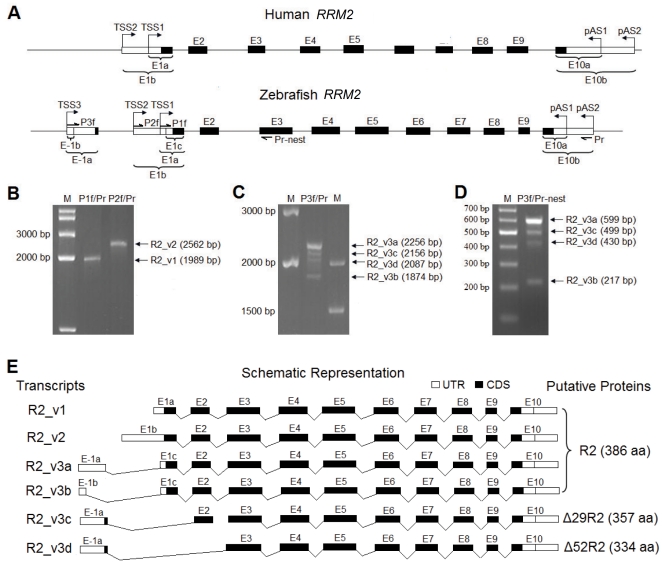
Genomic organization and transcript variants of zebrafish R2 gene. (**A**) Comparative analysis of R2 gene organization between human and zebrafish. Exons (E-1 to E10) are numbered and indicated by boxes. Solid boxes indicate the R2 coding region, whereas open boxes represent the 5′/3′-untranslated regions. Introns and other 5′-flanking regions are indicated by solid lines. Positions of primers used for RT-PCR are named and indicated by arrows. Alternative polyadenylation sites in exon 10 are shown as pAS1 and pAS2. (**B–D**) RT-PCR analysis of zebrafish R2 transcript variants. M: DNA size markers. (**E**) Schematic representation of zebrafish R2 transcript variants. Three distinct transcript variants named R2_v1, R2_v2 and R2_v3 are generated through alternative promoter usage. Alternative splicing of R2_v3 transcripts results in four transcript variants R2_v3a, R2_v3b, R2_v3c and R2_v3d. All six transcript variants contain E3 to E10. The three R2 forms are referred to as R2, Δ29R2 and Δ52R2.

To validate the *in silico* prediction of R2 transcript variants in zebrafish, RT-PCR assays were performed using primer pairs specific for the transcripts from three promoters (Table S1). A comparative analysis of genomic structures for R2 genes of human [33] and zebrafish demonstrated their difference in the numbers of TSSs, introns, exons and pASs. Forward primers P1_f, P2_f, and P3_f are located immediately downstream of three predicted transcriptional start sites of zebrafish R2 gene, respectively. A reverse primer P_r is located in the immediate vicinity of the pAS2. The primer nest-P_r was used for the nested-PCR (Figure 2A).

As shown in Figure 2B, RT-PCR assays with primer pairs P1_f/P_r and P2_f/P_r, which were designed to target the transcripts of promoter P1 and P2, produced a 1989-bp (R2_v1) and a 2562-bp (R2_v2) amplicon, respectively. Four amplicons from R2_v3a, R2_v3b, R2_v3c and R2_v3d were obtained using the primer pair P3_f/P_r (Figure 2C) and these amplicons were confirmed by a nested PCR with primers P3_f/P_r-nest (Figure 2D). As shown in Figure 2E, six types of R2 transcript variants contain exons 3 to 10, but exhibit significant differences at their 5′ termini. R2_v1 generated from promoter P1 contains a medium-length exon1 (E1a). R2_v2, the product of promoter P2, contains a long exon1 (E1b). The other four types of transcripts are products of promoter P3, including three alternative splicing variants (R2_v3b, R2_v3c and R2_v3d), and ESTs for R2_v3a and R2_v3c are found in the database of Genbank. Compared with R2_v1 and R2_v2, R2_v3 transcripts contain an extra E-1 with variations in length (E-1a and E-1b). R2_v3a, R2_v3c and R2_v3d contain a long exon-1(E-1a), whereas R2_v3b has a short exon-1(E-1b) from an alternative splicing donor site in E-1. R2_v3c derives from the skipping of E1c, whereas R2_v3d from the skipping of both E1c and E2. All splicing events occurred in six R2 transcript variants follow the “GU-AG” rule (data not shown).

Taken together, our results uncover six different transcript variants that derive from three predicated promoters and alternative splicing.

### Identification and characterization of three R2 promoters in HeLa cells and developing embryos

**Figure 3:**
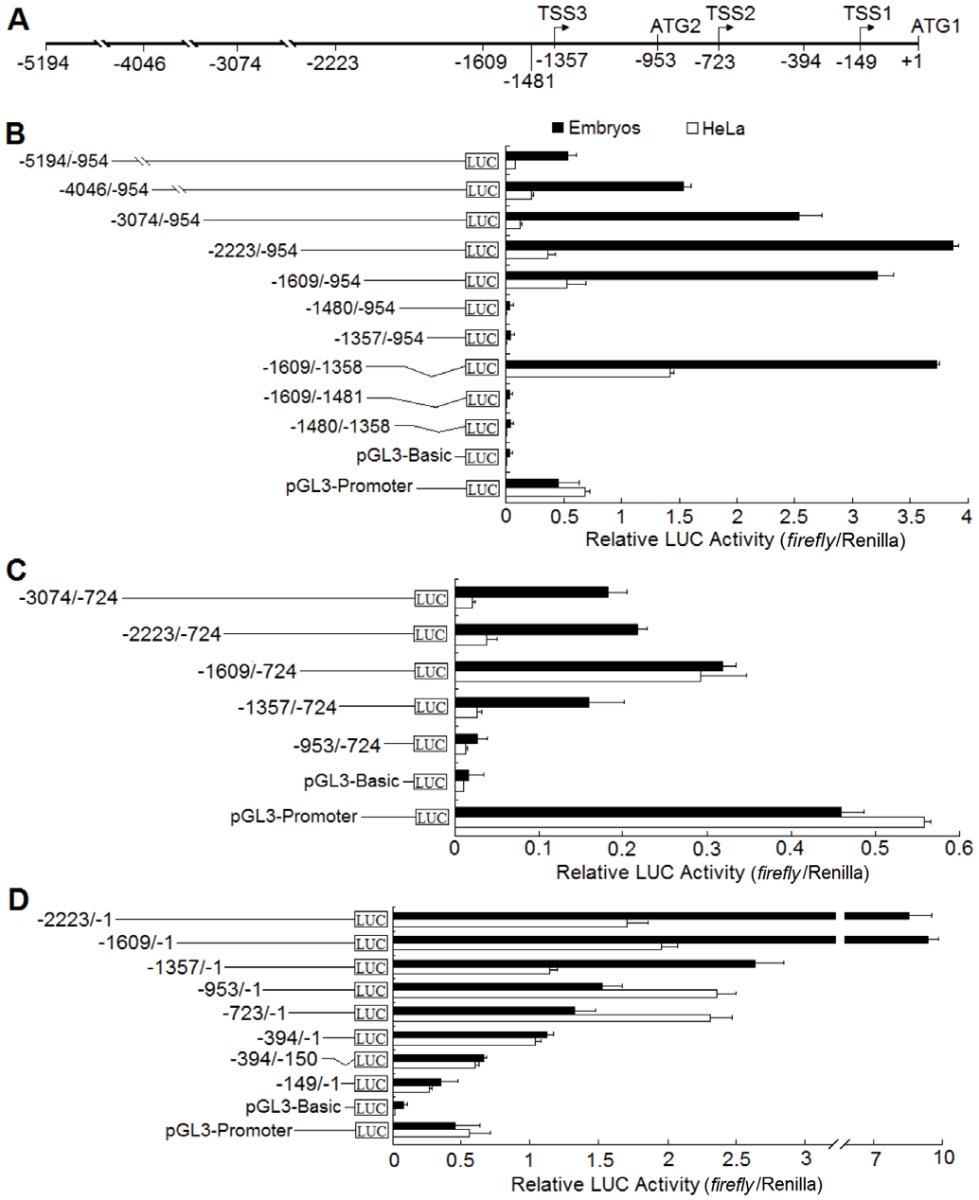
Analysis of the transcriptional regulatory region of the zebrafish R2 locus. (**A**) Genomic structure of the 5′ control region of R2 gene. The proximal translational initiation site (ATG) is designated as +1. Positions of two ATGs and three alternative transcriptional start sites (TSS1, TSS2 and TSS3) are shown. (**B–D**) Relative luciferase (LUC) activities (firefly/*Renilla*) of deletion constructs from three R2 promoters in zebrafish embryos and HeLa cells. Negative control pGL3-Basic, positive control pGL3-Promoter and promoter deletion constructs containing different lengths of the 5′-flanking region of R2 gene are listed in the left panel. Relative luciferase activities (firefly/*Renilla*) of corresponding constructs are presented in the right panel. Histograms represent means ± SD of three independent experiments.

To identify and characterize the three predicated R2 promoters, luciferase reporter assays were performed in HeLa cells and developing embryos. A 5.2-kb DNA fragment (-5194 to -1) of R2 gene was isolated from zebrafish genomic DNA (Figure 3A) and a series of promoter deletions from its termini were generated using primers listed in Table S1. These DNA fragments were then subcloned into the pGL3-Basic vector to drive the expression of luciferase reporter in transfected cells or microinjected embryos. The pGL3-Basic and pGL3-Promoter vectors were used as negative and positive controls.

To test the activity of the P3 promoter, ten promoter deletion constructs were made. As shown in Figure 3B, luciferase activities of four promoter regions (-4046/-954, -3074/-954, -2223/-954 and -1609/-954) were about 3 to 8-fold higher than those of SV40 promoter (pGL3-Promoter) in developing embryos. These results strongly suggest the presence of a predicated promoter P3. In addition, the activity of a DNA fragment (-1609/-1358, 245-bp immediately upstream of TSS3) was 2- or 8-fold higher than that of pGL3-Promoter in Hela cells and embryos. However, the activities of promoter region (-1609/-1481) and (-1480/-1358) were sharply decreased to the level of promoterless vector (pGL3-Basic). These data suggest that the DNA fragment (-1609 to -1358) contains the core sequence that is required for the basal activity of P3 promoter.

To investigate the activity of P2, five deletion constructs were made. As shown in Figure 3C, the activity of fragment (-953/-724) was nearly the same as that of pGL3-Basic, but activity of fragment (-1357/-724) in embryos was 10-fold higher than that of pGL3-Basic. These data suggest that the region (-1357/-724) harbors a minimal promoter of functional P2. Other three deletion fragments (-3074/-724, -2223/-724 and -1609/-724) exhibited higher luciferase activities than that of the fragment (-1357/-724), even though activities of all deletions were lower than that of pGL3-Promoter.

To detect the activity of the P1, eight promoter deletions were generated. As shown in Figure 3D, the luciferase activity of promoter region (-723/-1, a fragment between TSS2 and ATG1) showed 3- and 4-fold higher than that of SV40 promoter in both Hela cells and developing embryos, suggesting a functional P1 in zebrafish. Moreover, the promoter region (-394/-150, a fragment immediately upstream of TSS1), exhibited almost the same level of luciferase activity as that of pGL3-Promoter, indicating that this region contains a minimal promoter of P1. Since other promoter regions (-2223/-1, -1069/-1, -1357/-1 and -953/-1) contain elements of P2 and P3, luciferase activities of them were significantly higher than that of the promoter region (-723/-1).

Taken together, three functional promoters were characterized to drive alternative transcription of R2 gene in zebrafish and P1 appears to be the most active one.

### Spatiotemporal expression pattern of R2 transcript variants

**Figure 4:**
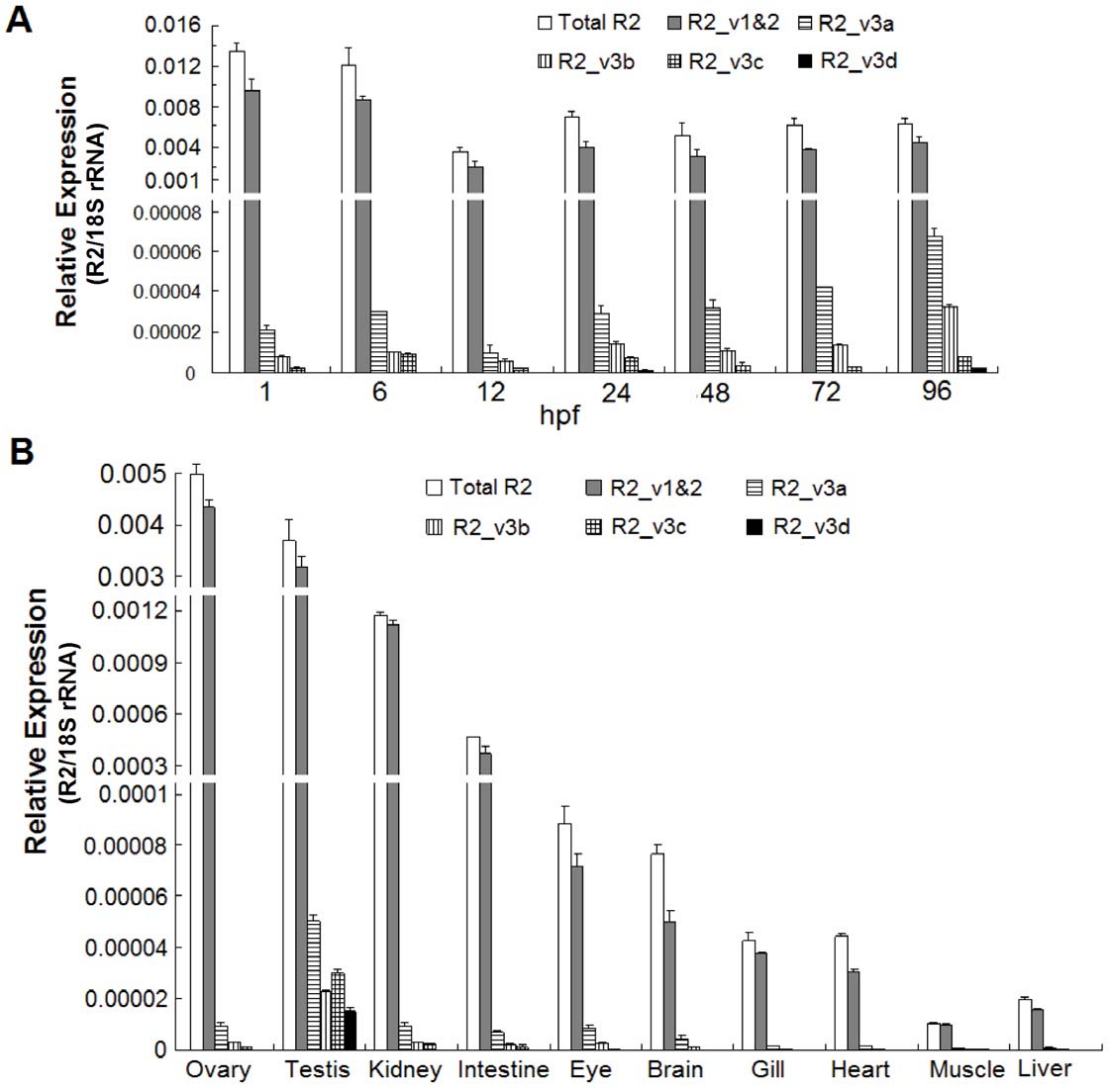
Spatiotemporal expression patterns of R2 transcript variants in zebrafish. Expression levels of R2 transcripts were detected by qPCR. (**A**) Expression of R2 transcripts during embryogenesis. Total RNA was isolated from thirty embryos at indicated stages. (**B**) Distribution of R2 transcripts in adult tissues examined. Total RNA was isolated from indicated tissues of two adult zebrafish. Expression levels of R2 transcripts were normalized to 18S rRNA expression, and the vertical bars represent the mean ± SD of three independent experiments.

To address the distribution of R2 transcript variants in developing embryos and adult tissues, quantitative PCR assays were performed. The data showed that high levels of total R2 transcripts including R2_v1 and R2_v2 were detected in early developing embryos at 1-6 hpf (Figure 4A) and in proliferating adult tissues including testis, ovary and kidney (Figure 4B). In comparison with R2_v3 variants, R2_v1 and R2_v2 were dominantly distributed in developing embryos and adult tissues. Moreover, ESTs for R2_v2 were not found in the GenBank database (data not shown) and activity of P2 is lower than P1. These data suggest that R2_v1 represents the vast majority of R2 transcripts and is highly expressed in proliferating cells.

In addition, R2_v3 were ubiquitously distributed in developing embryos and expressed at a high level in the late stage embryos (Figure 4A). However, the level of R2_v3 remains very low in most of tissues except testis (Figure 4B). Among four R2_v3 variants, R2_v3a is the dominant transcript variant in most of adult tissues examined and developing embryos at different stages (Figure 4A and 4B).

### S phase-specific expression of R2 gene in zebrafish

**Figure 5:**
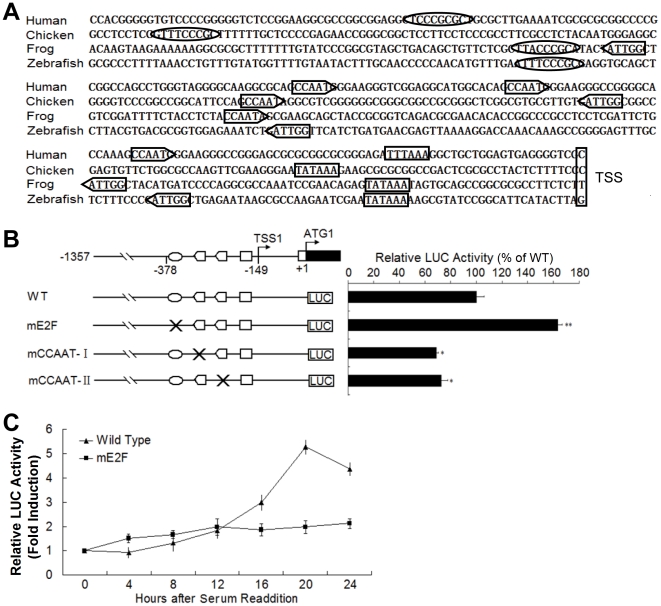
Functional analysis of E2F site and CCAAT box in the proximal promoter of zebrafish R2 gene. (**A**) Comparative analysis of 230-bp nucleotides upstream of the proximal TSS (indicated by vertical box) from human, chicken, frog and zebrafish. Predicated E2F binding site, CCAAT and TATA boxes are shown as ovals, arrows and rectangles, respectively. Accession numbers of these sequences were listed in Table S2. (**B**) Effects of mutations in the E2F binding site and CCAAT box on strength of proximal promoter in zebrafish R2 gene. Wild type (WT) and mutated promoters are indicated in the left panel and relative luciferase (LUC) activities of corresponding constructs are expressed as a percentage of wild type promoter activity in the right panel. ** and * indicate p<0.01 and p<0.05, respectively. (**C**) Effects of an E2F mutation on cell cycle-specific activation of zebrafish R2 promoter. HepG2 cells were transfected with wild type or mE2F reporter constructs plus reference vector pRL-SV40, synchronized by serum-starvation for 48 h and then stimulated by adding fresh DMEM with 20% FBS. Cells were harvested for luciferase assays at indicated time points. Values are expressed as fold induction compared with the relative luciferase activity (firefly/*Renilla*) at 0 h. Data represent mean ± SD from three independent experiments.

Since R2_v1 initiated by P1 appears to be preferentially expressed in proliferating cells, we next sought to determine molecular mechanism(s) underlying the regulation of R2_v1 expression. The sequences in the proximal regions of R2 promoters from zebrafish, frog, chicken and human were aligned. As shown in Figure 5A, a 230-bp DNA fragment immediately upstream TSS of the three R2 genes contains one TATA box (or its variant, TTTAAA), one E2F-binding site [34], and two (chicken and human) or three (zebrafish and frog) CCAAT boxes [35]. It is known that E2F-binding site and CCAAT boxes are essential for both basal and S phase-specific expression of mammal R2 [18]. To address whether these conserved elements in P1 of zebrafish R2 gene are required for the control of R2 expression during cell proliferation, three mutants of pGL-(-1609/-1) (mE2F, mCCAAT-I and mCCAAT-II) were generated via a PCR-based mutagenesis in the E2F-binding site or CCAAT box of wild type P1 (Left panel of Figure 5B). Then, effects of these mutations on P1 activity were detected in exponentially growing HepG2 cells. As shown in the right panel of Figure 5B, the mutation in E2F binding site led to an 65% increase in P1 activity (p<0.01), whereas mutations in CCAAT box I or II decreased P1 activity by 35% or 28%, respectively (p<0.05 in both cases). Thus, the E2F-binding site and CCAAT boxes are key *cis*-elements for the control of P1 activity in zebrafish. The E2F-binding site functions as a negative element, while CCAAT boxes serve as positive elements.

Since E2F-dependent repression is essential for cell cycle-specific expression of R2 gene in mouse [18], we then examined the negative effect of E2F binding site on expression of R2 gene in zebrafish. Transcriptional activities of wild type P1 and E2F mutant were determined in transiently transfected cells. Cells were synchronized by serum starvation followed by readdition of serum. It has been shown that the S phase duration of serum-deprived HepG2 cells is about 11–29 h after serum stimulation [36]. Our data showed that the luciferase activity of wild-type P1 markedly increased at 12 h and peaked at 20 h after serum stimulation, suggesting an S phase-specific induction; however, this effect was less pronounced for the mE2F construct (Figure 5C).

Taken together, our results indicate that the preferential expression of R2 gene in proliferating cells is associated with S phase-specific P1 activation that results from the relief of E2F-mediated repression.

### DNA damage-induced expression of R2 gene in zebrafish

**Figure 6:**
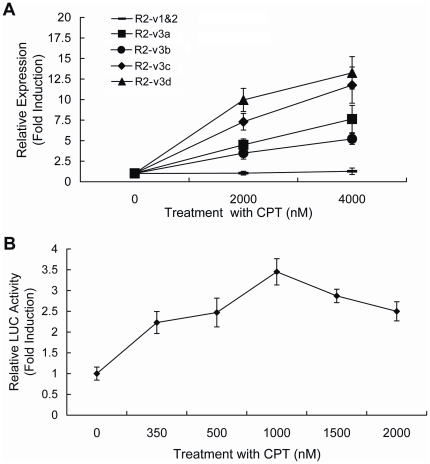
DNA damage-induced expression of zebrafish R2 gene. (**A**) Induced expression of R2 transcripts in CPT-treated embryos. Embryos at 24 hpf were treated with 2000 or 4000 nM CPT for 6 h and total RNA was isolated for real-time PCR. The relative expression levels of R2 transcripts are normalized to β-actin expression. Values are showed as the fold induction compared with untreated samples. (**B**) The activity of promoter P3 for zebrafish R2 gene was induced by CPT. The P3 pGL-(-5149/-954) reporter construct was co-injected with pRL-SV40 into one-cell stage embryos and injected embryos at 24 hpf were treated with indicated concentration of CPT for another 24 h. Then, luciferase (LUC) assays were performed with embryo lysates. Values are expressed as fold induction compared with untreated samples. All data represent means ± SD of three independent experiments.

Through evolution, expression of RNR subunit genes is tightly controlled in response to DNA damage [37] and their transcripts from multiple promoters or alternative splicing often exhibit distinct physiological implications [38]. To determine whether and which transcript variant of R2 gene in zebrafish is induced by DNA damage, expression of R2 gene in developing embryos treated with DNA damage reagents was investigated using real-time PCR. As shown in Figure 6A, treatment of developing embryos with 2 000 or 4 000 nM Camptothecin (CPT) led to a 3- to 13-fold increase in the levels of four R2_v3 transcripts that are derived from P3 promoter. Levels of R2_v3c and R2_v3d increased 11- and 13-fold, respectively; however, R2_v1&2 levels were nearly unaffected. To further determine whether DNA damage reagent could induce expression of R2_v3 at the level of transcription, luciferase activity of pGL-(-5194/-954) in CPT-treated embryos was tested. As shown in Figure 6B, the activity of P3 was induced by certain concentrations of CPT in a dose-dependent manner. These results suggest that R2_v3 transcripts are specifically induced by DNA damage signals and that this inductive effect is closely associated with the transcriptional activation of P3.

### Protein isoforms derived from zebrafish R2 transcript variants

As shown in Figure 2E, three R2 isoforms were deduced from six transcript variants derived from alternative promoters and splicing. R2_v1, R2_v2, R2_v3a and R2_v3b encode the normal subtype of R2, which resembles mammal R2. R2_v3c and R2_v3d are generated by skipping of exon 1c (E1c) and exon 1c&2 (E1c& E2), and translated from an alternative translation start site at nucleotide position -950 (Figure 1). As a result, R2_v3c and R2_v3d encode two N-terminally truncated forms of R2, which will hereinafter be referred to as Δ29R2 and Δ52R2.

**Figure 7:**
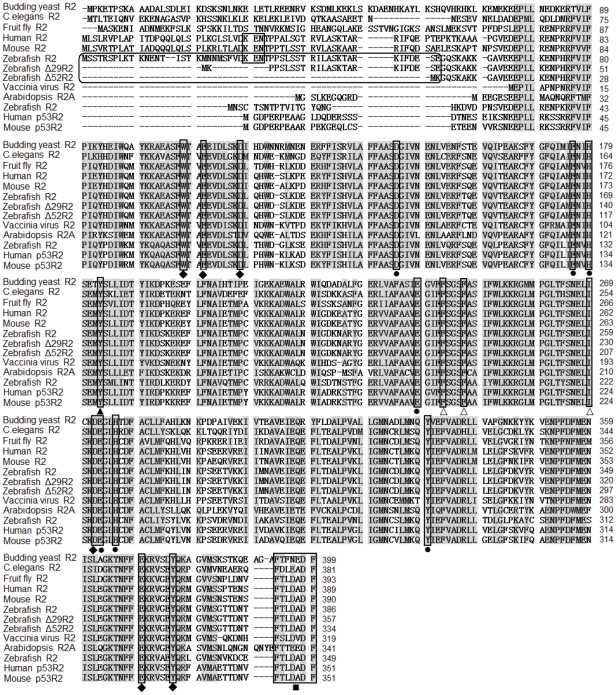
Multiple sequence alignments of RNR small subunits from nine species. Gaps were introduced to maximize the alignment. Amino acids with a similarity of more than 80% are shadowed. The KEN boxes are indicated by rectangle. The N-termini of three zebrafish R2 isoforms is indicated by rounded rectangle. Residues crucial for enzyme activity are boxed and indicated by different symbols: • =  iron ligands; ▴ =  tyrosyl radical; Δ = hydrophobic pocket; ♦ = electron transport; ▪ =  R1 binding heptapeptide. Accession numbers of these sequences were listed in Table S2.

To further analyze the functional difference among three R2 isoforms in zebrafish, multi-alignments of RNR small subunits from many species were performed. As shown in Figure 7, all of R2 isoforms in zebrafish contain most of the residues that are essential for RNR enzyme activity and are conserved in RNR small subunits from different species. These residues are necessary for iron ligands, tyrosyl free radical generation, formation of hydrophobic pocket surrounding the radical, electron transport and C-terminal heptapeptide binding to the R1 protein [39], [40]. In particular, the KEN box that mediates the degradation of mammalian R2 outside of the S phase [20] is conserved in the normal form of zebrafish R2, whereas both Δ29R2 and Δ52R2 lack this functional domain at their N-terminus.

### Isoforms of zebrafish R2 are localized in the cytosol and physically interact with R1

**Figure 8:**
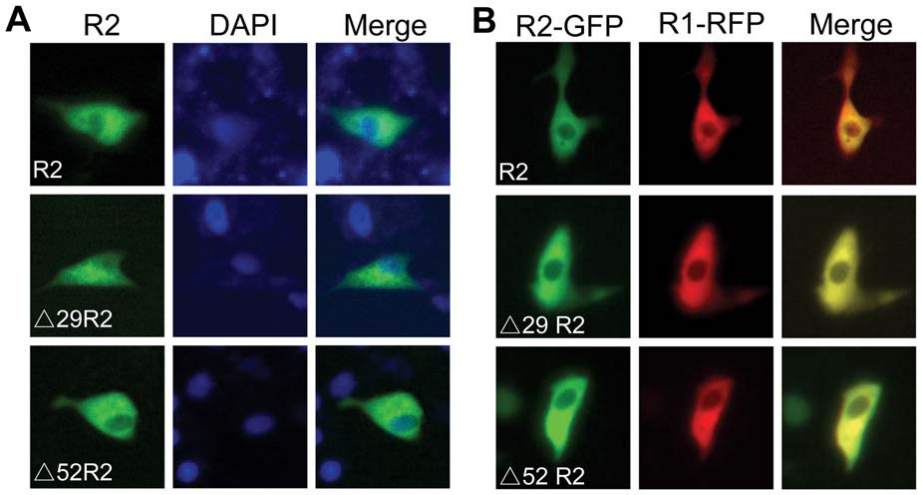
Subcellular localization of zebrafish R2 isoforms. (**A**) Immunofluorescence staining was performed to detect the subcellular distribution of zebrafish R2 isoforms in transfected Hela cells. Three R2 isoforms were tagged with a Flag at N-terminus. At 36 h after transfection, R2 isoforms were detected with primary anti-Flag antibody and FITC-conjugated secondary antibody. Nuclei were stained with DAPI. (**B**) Co-localization of fluorescent protein-tagged zebrafish R1 and R2 isoforms in the cytosol of Hela cells. R2 isoforms or R1 of zebrafish were fused with GFP or RFP to their C-termini. At 36 h after transfection, images were directly acquired under fluorescence microscopy.

Since subcellular distribution of RNR subunits play crucial roles in the regulation of RNR activity, we investigated the localization of the three putative R2 isoforms in transfected Hela cells. As previously described [41], the coding sequences of three R2 isoforms and R1 were tagged with Flag, HA, GFP or RFP. Immunofluorescence staining assays indicated that three isoforms of zebrafish R2 were mainly distributed in the cytoplasm of Hela cells (Figure 8A). Moreover, GFP-tagged R2 and RFP-tagged R1 were co-localized in the cytosol of Hela cells (Figure 8B).

**Figure 9:**
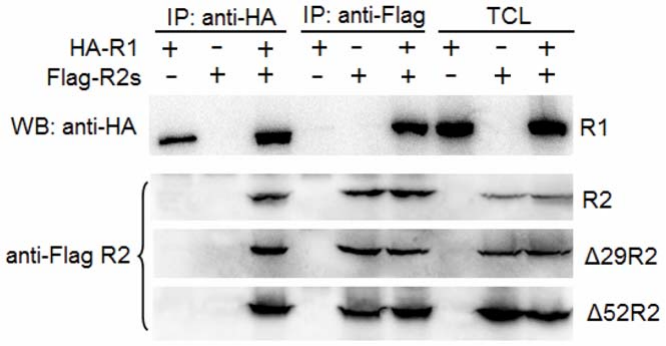
Physical interaction of zebrafish R2 isoforms with R1. 293T cells were transfected with vectors expressing HA-R1 and one of Flag-R2 isoforms. At 45 h after transfection, protein extracts were subjected to immunoprecipitation (IP) with anti-HA or anti-Flag antibodies. IP materials and total cell lysates (TCL) were detected by Western blotting (WB) analysis. Data for R1 are representative of three IP samples.

Next, we addressed whether N-terminally truncated R2 isoforms are able to associate with R1. HA-tagged R1 and one of the Flag-tagged R2 isoforms were co-expressed in transfected HEK293T cells. Co-immunoprecipitation and Western blotting assays were then conducted with monoclonal antibodies against Flag or HA. As shown in Figure 9, Δ29R2 and Δ52R2 can be precipitated with HA-tagged R1 and detected using the anti-Flag antibody, while R1 can be precipitated with either Flag-tagged Δ29R2 or Δ52R2 and detected using the anti-HA antibody. These results suggest that N-terminally truncated isoforms of zebrafish R2 are able to physically interact with R1.

## Discussion

RNR subunits are highly conserved during evolution and their expression is tightly controlled by multiple mechanisms [1]. However, it remains largely unknown about regulation and functions of RNR subunits in zebrafish. A transcript encoding the normal form R2 in zebrafish has been identified without characterization of its functions [30]. We have recently shown that expression and functions of p53R2 in zebrafish are closely associated with its activities in DNA repair and synthesis [31]. In this study, we demonstrate intrinsic mechanisms underlying the control of zebrafish R2 expression, including alternative promoter usage, pre-mRNA splicing and polyadenylation site selection. Six distinct transcripts that are derived from three promoters are characterized to encode three R2 isoforms. Transcripts of normal R2 is mainly expressed in a cell cycle-specific manner, while transcripts of Δ29R2 and Δ52R2 are induced by DNA damage. Our results provide new evidence for the tight control of differential expression and functions of R2.

### Regulation of R2 gene expression by alternative promoters in zebrafish

It has been shown that the use of alternative promoters is prevalent in many eukaryotic genes [42] and orthologs of R2 genes in human and fission yeast harbor two promoters with distinct transcriptional activities [33]. In this study, we have identified three functional promoters of R2 gene in zebrafish. These three promoters are able to generate six different transcripts with different 5′ termini. In accordance with previous studies on functions of R2 genes in other species [43], [44], our data from quantitative PCR and characterization of P1 activity indicate that zebrafish R2 gene is preferentially expressed in proliferating and dividing cells. Activity of P1 mainly generates a transcript variant of R2_v1 in an S phase-specific manner. Similar to those in human and mouse [19], [33], [45], the CCAAT box in zebrafish P1 is required for the promoter strength, while the E2F-binding site is indispensable for the S phase specificity. Additionally, it is shown that the E2F-binding site, identified as E2F4 in mouse R2 gene [18], functions as a marginal transcriptional repressor [46]. Interestingly, the E2F-binding site in higher plants also plays a crucial role in cell cycle-specific transcription of R2 homologous gene [11], [16]. Thus, E2F repression appears to be a conserved mechanism underlying the cell cycle-specific transcription of R2 genes in high plants and vertebrates.

Transcription of RNR small subunit genes in many species including social amoeba [47], yeast [48], [49] and higher plants [16], can be induced by DNA damage signals. In this study, we demonstrate that the expression of four R2_v3 tanscript variants are induced by DNA damage regents and this inductive effect is closely associated with the differential activation of P3, which leads to a 3-13 fold induction of R2_v3 variant. It is shown that homologues of Crt1/Rfx1 which work as transcription repressors play an important role in for DNA damage induced transcription of R2 gene in yeast and mammalian cancer cells [50]. Furthermore, E2F factors are also involved in the DNA damage-induced expression of the R2 gene in human tumors [51], particularly E2F sites directly mediates this induction effect in plants [11], [16]. Interestingly, the P3 of zebrafish R2 gene contains binding sites for E2F, Rfx1 and other transcription factors Oct-1 and AP-1 (data not shown), which are known to be involved in the regulation of stress-induction [52]. Therefore, further efforts are needed to address mechanism(s) underlying the induced expression of R2 gene upon DNA damage.

### Regulation of R2 gene expression by alternative splicing and polyadenylation in zebrafish

Alternative promoters can initiate transcription from different exons and tend to generate alternative splicing which is a widespread mechanism of gene regulation in higher eukaryotes [53]. In this study, we have identified six R2 transcript variants with distinct 5′ termini in zebrafish. Three of the transcript variants generated by P3 are derived from exon skipping and usage of alternative splice donor sites. Furthermore, we demonstrate that transcript variants from P3 promoter are differentially induced by DNA damage reagents. This observation is consistent with previous studies showing that alternative splice sites can be selected by cells responding to extracellular signals [54]. However, it remains unclear how the activity and specificity of the splicing machine is controlled by DNA damage signals.

Alternative polyadenylation is another mechanism that yields transcripts with identical protein-coding sequences and different 3′ UTRs, which provides the potential for differential regulation of mRNA expression by RNA binding proteins and/or miRNAs [55]. Two functional polyadenylation sites and several conserved *cis*-elements are found in the 3′ untranslated region (3′ UTR) of zebrafish R2 gene (Figure S1). The proximal polyadenylation signal is likely required for the abundant expression of zebrafish R2 gene during early embryonic development and in reproductive tissues since it exists in most of ESTs from the GenBank database (Figure S1). In addition, a cytoplasmic polyadenylation element that mediate the maternal expression of R2 gene in sea urchin egg [56] is found near the proximal polyadenylation site of zebrafish R2, and shorter 3′ UTRs are usually associated with cell proliferation [57]. Moreover, the distal polyadenylation signal appears to link with DNA damage-induced expression of R2 gene, since eight AU-rich elements are found in the 3′ UTR between two polyadenylation signals in zebrafish R2 gene. These AU-rich elements are well known to target mRNAs for rapid degradation and their presence can lead to the stabilization of a mRNA depending on precise stimulus [58].

### The putative isoforms of zebrafish R2 are catalytically active

Most of alternative splicing events can lead to the synthesis of different protein isoforms because of alterations in their coding region [59]. In this study, we show that alternative splicing of R2_v3 transcripts give rise to three R2 isoforms: one normal R2 and two novel R2 isoforms truncated at N-terminus (Δ29R2 and Δ52R2). Although alterations in the sequence of proteins can affect their binding properties, subcellular localization, enzymatic activity and/or stability [60], our *in vitro* data indicate that N-terminal truncations of zebrafish R2 isoforms didn′t alter their cytoplasmatic localization and interaction with R1.

Several lines of evidence suggest that the N-terminal region of vertebrate R2 is dispensable for its catalytic activity. First, amino acid residues at the N-terminus of R2 genes from different species are not conserved and their N-terminal regions differ in length. For instance, N-terminal regions of R2 are missed in large DNA viruses [61], protozoan parasites [62], higher plants [13], and *Escherichia coli* [63]. In mammals, the major difference between R2 and p53R2 is that the latter lacks 33 residues in its N-terminus [23]. Second, the structural biology of mouse R2 indicates that 65 residues at its N-terminus are disordered and thus not visible in the crystal structure [64]. Third, a recombinant mouse R2 protein lacking the N-terminal 61 residues is able to interact with the R1 and is fully active *in vitro* [20]. Vaccinia R2 lacking the N-terminal 65 residues interacts with mouse R1 to form active complexes *in vivo* [65]. Thus, two N-terminally truncated isoforms of zebrafish R2 are likely to have the catalytic activity.

The N-terminal region of R2 appears to be important for cell cycle-specific regulation of R2 expression. It is shown that residues 30-32 in the KEN box of mouse R2 mediate its mitotic degradation and these N-terminal regulatory sequences are conserved among R2s from metazoan, *C. elegans* and fruit fly [20]. The normal form of R2 in zebrafish contains a KEN box, whereas two N-terminally truncated isoforms (Δ29R2 and Δ52R2) lose it. It is likely that the ingenious truncation in zebrafish R2 results in active and stable forms of R2 throughout the cell cycle.

### Zebrafish R2 gene has a redundant function, overlapping with p53R2 in response to DNA damage

RNR functions in supplying dNTPs for DNA synthesis and DNA repair and organisms have developed complicated mechanisms throughout evolution to control the differential expression of RNR subunit genes. The single R2 in lower animals (Figure S2) possesses two distinct functions: S phase-specific expression for DNA replication and DNA damage-induced expression for DNA repair. In vertebrates, it is likely that a subfunction partitioning has occurred during the evolution of R2 genes, since R2 and p53R2 encoded by two different genes attribute to the functions of the RNR small subunit. R2 is exclusively responsible for nuclear DNA replication, whereas p53R2 functions in DNA repair and mitochondrial DNA replication [66]. In association with this subfunctionalization, R2 is expressed in an S phase-specific manner and is degraded during mitosis through the N-terminal KEN box [20], whereas expression of p53R2 is induced by DNA damage signals in a p53-dependent manner [23]. p53R2 contains no KEN box and it is stabilized after DNA damage [25]. We have recently demonstrated that zebrafish p53R2 can be induced by DNA damage reagents and plays conserved functions in genotoxic stress [31]. In this study, we show that two p53R2-like R2 isoforms are generated through alternative promoter usage and pre-mRNA splicing in zebrafish. These observations are consistent with previous studies showing that alternative promoter usage and splicing of R2 genes in fission yeast and mosquito occur in response to DNA damage [49], [67]; however, there is no p53R2-like gene in these species. Additionally, two N-truncated R2 isoforms in zebrafish are strongly associated with DNA damage response, whereas truncated R2 isoforms have yet to be characterized in mammals. Therefore, the R2 gene in zebrafish appears to have a redundant, overlapping function with p53R2 in response to DNA damage. Further studies are needed to address whether p53R2 and R2 isoforms have differential functions under genotoxic stress in zebrafish.

## Materials and Methods

### Ethics statement

The animal protocol for this research was approved by the Animal Care and Use Committee of Hubei Province in China and by the Institutional Animal Care and Use Committee of Institute of Hydrobiology (Approval ID: Keshuizhuan 0829).

### Bioinformatic analysis

Putative promoters of R2 gene were analyzed using the Promoter Scan (http://www-bimas.cit.nih.gov/molbio/proscan/), transcription factor binding sites were predicated using the Genomatix suite (http://www.genomatix.de/) and TFSEARCH (http://www.cbrc.jp/research/db/TFSEARCH.html). cDNA/EST sequences were obtained from the UniGene database. Intron/exon structures were determined through a comparison of cDNA with the corresponding genomic sequence using the Spidey software (http://www.ncbi.nlm.nih.gov/spidey/). Transcriptional start sites were predicted using the Eponine Transcriptional start Site Finder (http://servlet.sanger.ac.uk.8080/eponine/). Alignment of R2 proteins from different species was performed using the ClustalW2 (http://www.ebi.ac.uk/Tools/clustalw2/index.html).

### Zebrafish and chemical mutagens

AB inbred strain of zebrafish were raised and maintained under standard conditions. Naturally fertilized zebrafish embryos were incubated at 28°C, and staged by hours post-fertilization (hpf).

Camptothecin (CPT) and methylmethane sulfonate (MMS) were purchased from Sigma-Aldrich. Stock solution of CPT or MMS at 10 mM was prepared in dimethyl sulfoxide (DMSO), stored at −20°C and diluted to desired concentrations immediately prior to usage.

### Cell lines and transient transfection

HeLa, HepG2 and 293T cells (ATCC Numbers: CCL-2, HB-8065, CRL-11268) were maintained in Dulbecco′s modified Eagle′s Medium (DMEM) supplemented with 10% fetal calf serum (FCS), 100 u/mL penicillin, 100 µg/mL streptomycin and 0.25 µg/mL fungizone from Invitrogen, at 37°C in 5% CO_2_/air atmosphere. Transfection was carried out using the FuGENE 6 reagent from Roche according to the manufacturer′s instructions. The total DNA amount used for each transfection was kept constant by adding the appropriate amount of parental empty expression vector. Serum-starved cells were prepared from the culture of HepG2 in medium with 0.5% FCS for 48 h and then stimulated by adding fresh DMEM with 20% FBS [68].

### RNA extraction, RT-PCR and quantitative PCR

Total RNA was extracted from about 35 developing embryos or adult tissues from 2-3 individuals using the TRIZOL reagent (Invitrogen). RNA samples were digested with RNase Free DNase I (Promega). The RNA integrity and quality were then determined by agarose electrophoresis and spectrophotometer. The cDNAs were transcribed from 2 µg of total RNA using the RevertAid™ First Strand cDNA Synthesis Kit (Fermentas) in a reaction volume of 20 µl. The reaction conditions and thermal profile were set up according to the instructions of the MyiQ Single-Color Real-Time PCR Detection System from Bio-Rad.

For reverse transcription PCR (RT-PCR) analysis, mixture of cDNAs (primed with oligo-dT_18_) from testis and ovary were used as template using specific primer pairs listed in Table S1. PCR products were analyzed on 2% agarose gel and sequenced.

For real-time quantitative PCR (qPCR), random hexamers were used for cDNA synthesis because the oligo-dT primers are not suitable for examination of splice variants and 18S ribosomal RNA (rRNA). Six validated primer pairs (Table S1) were manually designed to specifically target R2 splice variants. To avoid amplifying genomic DNA, each primer pair contains at least one primer spanning an “exon-exon” boundary. The primer pair v1&2_f/v1&2_r was used to detect two transcripts R2_v1 and R2_v2, due to a little difference in their exon1 sequence that does not allow the design of another primer pair to distinguish between them. The four v3_f/v3_r primer pairs were used to amplify four variants of R3_v3 subclass specifically. The primer pair total_f/total_r was designed to detect the total level of R2 transcripts. All of the PCR products were sequenced. The 18S rRNA was used as the reference to calculate the relative quantification of R2 transcripts in developing embryos and adult tissues. The β-actin was used as the reference to detect the relative quantity of R2 expression upon DNA damage according to a previous study [69].

The qPCR assays were performed using the MyiQ Single-Color Real-Time PCR Detection System from Bio-Rad in a reaction volume of 20 µl containing 5 µl of diluted (1∶10) cDNA, 100 nM of each primer and 10 µl of the 2×SYBR Green I Master Mix (Toyobo). Reaction conditions are as follows: 1 cycle at 95°C for 3 min; 40 cycles at 95°C for 10 s and 60°C for 30 s. All samples were run in triplicate. No template controls (NTC) were included in all of qPCR assays and did not show any amplification. After amplification, melting curve analysis was performed to avoid the existence of other nonspecific products including primer dimmers and unintended amplification of genomic DNA. The specificity of PCR products was further confirmed by electrophoresis and sequencing. Amplification efficiency of each primer pair was calculated using the corresponding standard curve, which was obtained by plotting cycle threshold (Ct) values against log-transformed serial ten-fold dilutions (Figure S3). No detectable Ct values were obtained from NTCs. Efficiencies of primer pairs for 18S rRNA, β-actin, total R2, R2_v1&2, R2_v3a, R2_v3b, R2_v3c and R2_v3d are 100.94%, 100.96%, 97.32%, 97.83%, 98.95%, 100.49%, 97.92 and 101.16%, respectively. These data meet the requirements for analysis of raw data with the 2^-ΔΔCT^ method [70] in the application guide of manufacturer (Bio-Red, Catalog # 170-9799).

### Generation of DNA constructs

To generate promoter deletion constructs, a primer pair -4046_f/-1_r was designed to amplify a 4-kb DNA fragment from the 5′ control region of zebrafish R2 gene according to an annotated sequence in GenBank (BX248136). This fragment was inserted into the pGL-Basic vector from Promega. Promoter deletion constructs for P1 (-3074/-1, -2223/-1, -1609/-1, -1357/-1, -953/-1, -723/-1, -394/-1, -149/-1, -394/-150), P2(-2223/-724, -1609/-724, -1357/-724, -953/-724) and P3(-5194/-954, -4046/-954, -3074/-954, -2223/-954, -1609/-954, -1480/-954, -1357/-954, -1609/-1358, -1609/-1481, -1480/-1358) were then generated with PCR primers listed in Table S1.

To generate promoter mutants, a megaprimer PCR approach was used [71]. Primer pair -1357_f /-1_r was used as the flanking primers. Three “megaprimers” were listed in Table S1. The E2F binding site sequence TTTCCCGCG was changed to TTTCCTCAT [18] and the CCAAT box sequence was substituted with the CTAGT [72].

To construct vectors for ectopic expression of R1 and R2, the coding sequence of R1 gene was inserted into the vector pCGN-HAM [73] and pDsRed1-N1 from Clontech, and coding sequences for three putative R2 isoforms were inserted into the pCMV-Tag2c and pAcGFP-N1 from Clontech, respectively. All constructs were confirmed by sequencing.

### Luciferase assays

To analyze the activity of R2 promoter deletions, luciferase reporter vector plus the reference pRL-TK were co-transfected or co-microinjected into HeLa cells or developing embryos. At 48 h after transfection or microinjection, samples were harvested for luciferase assays.

To analyze the activity of promoter mutants, luciferase reporter vectors containing one of promoter mutants were transfected into HepG2 cells, which are more suitable for serum starvation arrests than HeLa cells [74]. pRL-SV40 was used as the reference as previously described [18]. Samples were collected at 48 h post-transfection. To determine the effect of serum stimulation on R2 promoter, HepG2 cells were synchronized using the serum starvation–stimulation protocol [36]. Once transfections were completed, cells were maintained in DMEM containing 0.5% FBS for 48 h. The medium was then changed to DMEM containing 20% FBS and cells were harvested at different time points.

To detect effects of DNA damage on the activity of R2 promoter, the normal or E2F-mutant promoter was microinjected with pRL-SV40 into one-cell stage embryos. At 24 h after injection, embryos were exposed to 0, 1000, 2000, or 3000 nM CPT for another 24 h and then 30 developing embryos in each group were collected for luciferase assays.

The luciferase activity was quantified in an analytical Luminometer from Berthold using the Dual-Luciferase Reporter Assay System from Promega. Data were expressed as the ratio of firefly to Renilla luciferase activity.

### Immunofluorescence staining, co-immunoprecipitation and Western blotting

HeLa cells were transfected with one of the constructs expressing three putative isoforms of R2 tagged with Flag. At 24 h after transfection, immunofluorescence staining assays were performed following our previous protocol [75]. Subcellular co-localization of RFP-tagged R1 and GFP-tagged R2 was directly visualized in HeLa cells under a fluorescence microscope from Nikon. Physical interaction of three R2 isoforms with R1 was detected in transfected 293T cells following our previous protocol [75].

## Supporting Information

Figure S1 **Alternative polyadenylation sites of R2 gene in zebrafish.** (**A**) Two functional polyadenylation sites (pAS) of zebrafish R2 gene were found through bioinformatics analysis of cDNA/ESTs with polyadenylation signals in the UniGene database. Only non-normalized and non-subtracted EST libraries were considered, so the numbers of ESTs given for each site were taken as a measure of relative polyadenylation efficiency. (**B**) Nucleotide sequence of the 3′ untranslated region in R2 gene of zebrafish. Consensus sequences of polyadenylation signals, upstream sequence elements (USE) and AU-rich elements (ARE) crucial for mRNA stability were indicated. The sequence of R2 gene is shown in upper case, while the 3′ flanking genomic sequence is shown in lower case.
(TIF)

Figure S2 **Phylogenetic analysis of class I a RNR small subunits.** The phylogenetic tree was inferred using the Neighbor-Joining method and phylogenetic analysis were conducted in MEGA4. Numbers at nodes represent percentage bootstrap values obtained from 1,000 samplings. R2s which are reported to be induced by DNA damage are indicated. Accession numbers of these sequences were listed in Table S2.
(TIF)

Figure S3 **Standard curves and amplication efficiencies of primer pairs used for qPCR.** (A) 18S rRNA. (B) β-actin. (C) Total R2. (D) R2_v1&2. (E) R2_v3a. (F) R2_v3b. (G) R2_v3c. (H) R2_v3d.
(TIF)

Table S1 Primers used in this study.
(DOC)

Table S2 Accession numbers of sequences used in this study.
(DOC)
