# Supplementary figures and images for: Identification and Characterization of Alternative Promoters, Transcripts and Protein Isoforms of Zebrafish R2 Gene
Source: PLoS One. 2011 Aug 24;6(8):e24089. doi: 10.1371/journal.pone.0024089 (PMC3161108; doi:10.1371/journal.pone.0024089)

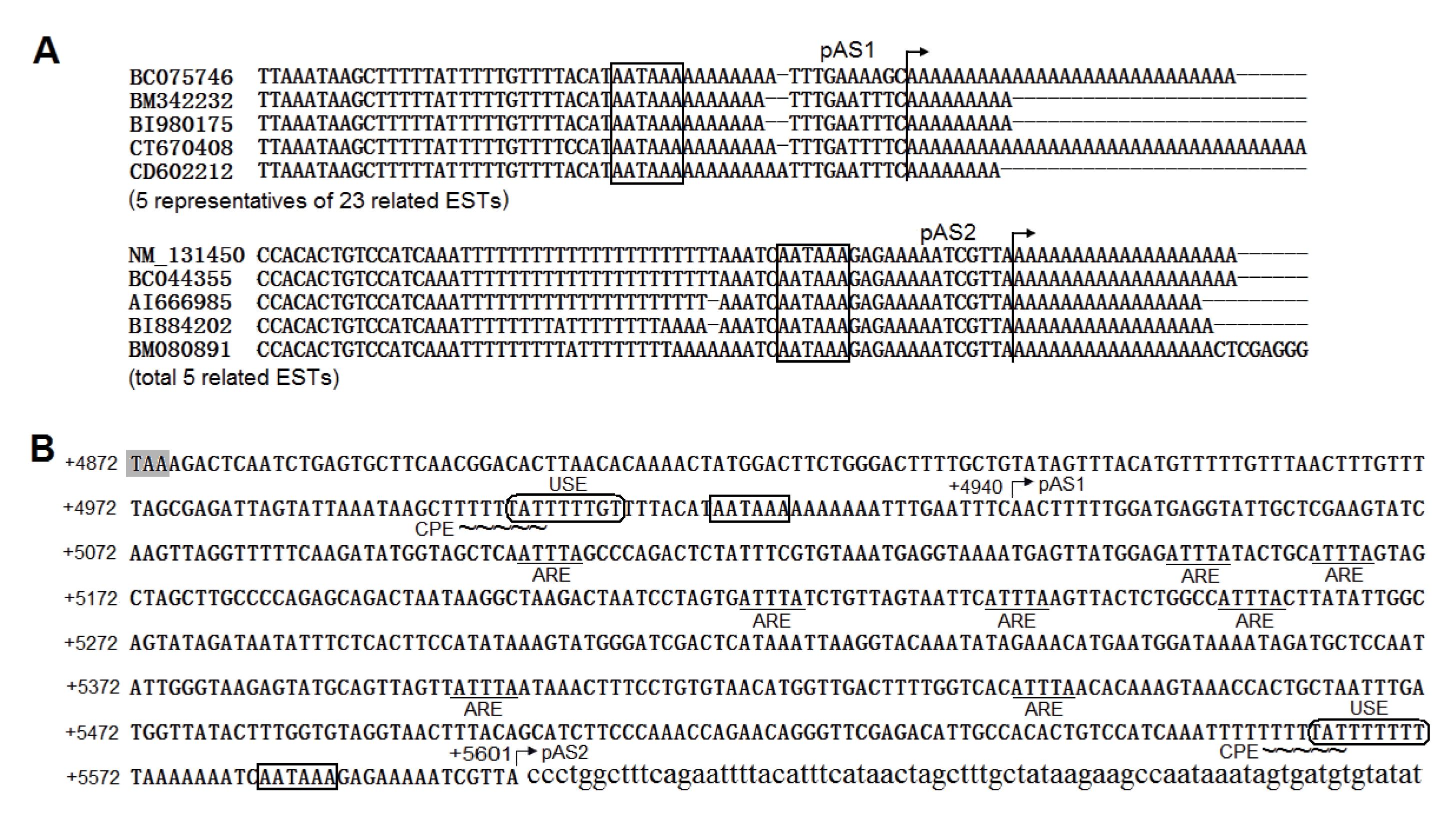

Supplement: Figure S1 — Alternative polyadenylation sites of R2 gene in zebrafish. (A) Two functional polyadenylation sites (pAS) of zebrafish R2 gene were found through bioinformatics analysis of cDNA/ESTs with polyadenylation signals in the UniGene database. Only non-normalized and non-subtracted EST libraries were considered, so the numbers of ESTs given for each site were taken as a measure of relative polyadenylation efficiency. (B) Nucleotide sequence of the 3′ untranslated region in R2 gene of zebrafish. Consensus sequences of polyadenylation signals, upstream sequence elements (USE) and AU-rich elements (ARE) crucial for mRNA stability were indicated. The sequence of R2 gene is shown in upper case, while the 3′ flanking genomic sequence is shown in lower case. (TIF) [file pone.0024089.s001.tif]

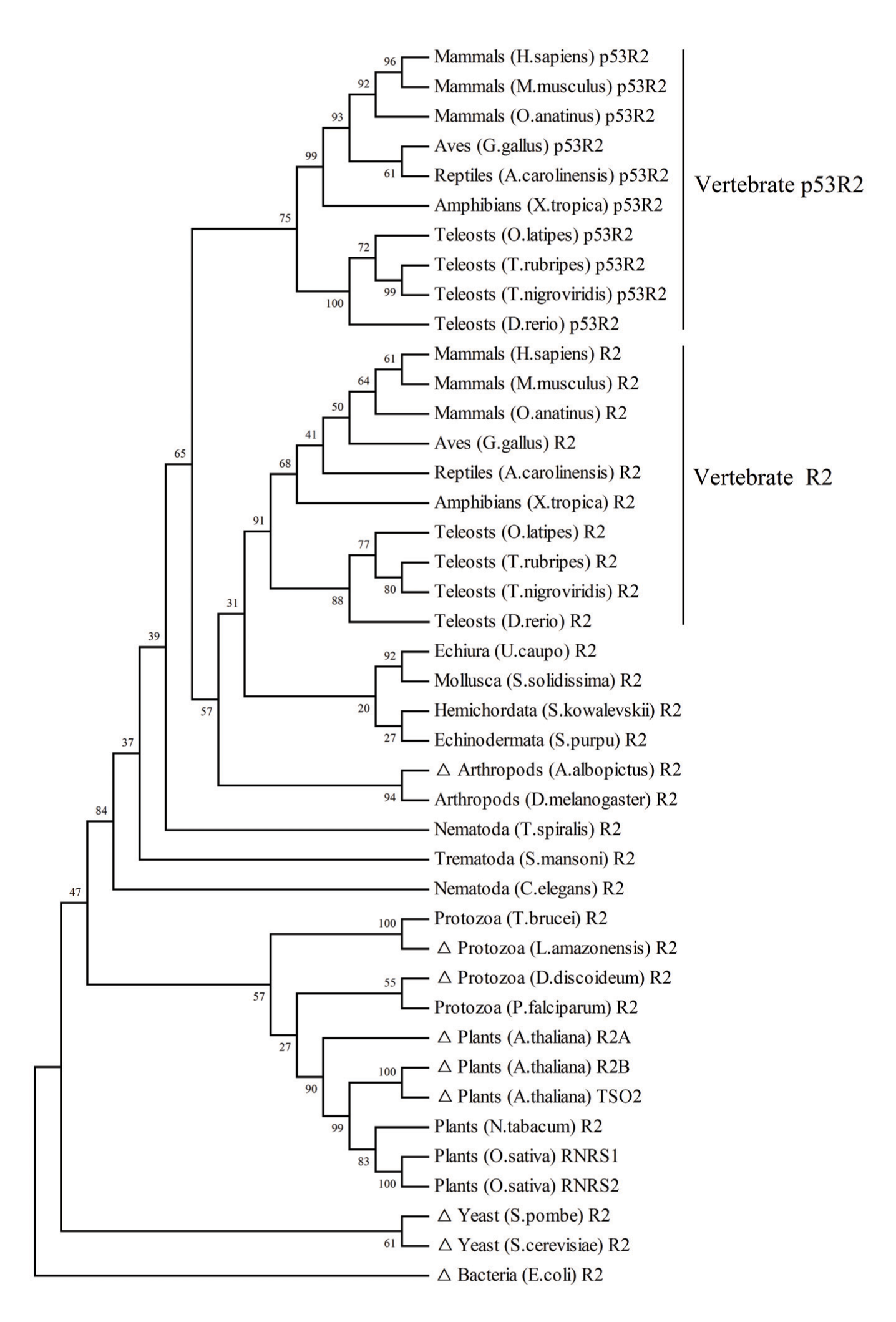

Supplement: Figure S2 — Phylogenetic analysis of class I a RNR small subunits. The phylogenetic tree was inferred using the Neighbor-Joining method and phylogenetic analysis were conducted in MEGA4. Numbers at nodes represent percentage bootstrap values obtained from 1,000 samplings. R2s which are reported to be induced by DNA damage are indicated. Accession numbers of these sequences were listed in Table S2. (TIF) [file pone.0024089.s002.tif]
