# Supplementary material for: Identification and Characterization of Alternative Promoters, Transcripts and Protein Isoforms of Zebrafish R2 Gene
Source: PLoS One. 2011 Aug 24;6(8):e24089. doi: 10.1371/journal.pone.0024089 (PMC3161108; doi:10.1371/journal.pone.0024089)
Supplement: Table S1 — Primers used in this study. (DOC) [file pone.0024089.s004.doc]

**Table S1. Primers used in this study**

| **Used for** | **Name** | **Sequence (5’-3’)** |
| --- | --- | --- |
| **Promoter deletion** | -5194_f | TGTCAGGTGTTTCCTGTTAAC |
|  | -4046_f | GCGATACTACCTGCAATATTC |
|  | -3077_f | TATCTCGAG TAACGCAAGAACTTGGCGATG |
|  | -2226_f | TGTCTCGAGGCGCGCATACATGCATG |
|  | -1611_f | TGTCTCGAGTAATTCAGCTCTTGACATAAG |
|  | -1480_f | TGTCTCGAGGATAAATTAATAATG |
|  | -1359_f | TATCTCGAGGACTGTGACCTCTTGGGC |
|  | -955_f | TATCTCGAGATGAAGGTATAATCAC |
|  | -725_f | TATCTCGAGCCCAAACAACAGTCGATTG |
|  | -396_f | TATCTCGAGGTTTTATTTCATATTCG |
|  | -150_f | TATCTCGAGGTGTTATCTTCGCTTC |
|  | -1_r | TA GGATCCTGTAAAGCTTGGAGTGTTTGG |
|  | -956_r | ATGGATCCCAAATCCTCCGTATGA |
|  | -726_r | ATGGATCCGAAATACATTTCTCCG |
|  | -2227_r | TAGGATCCGCCTGTTGACCTTTTG |
|  | -1360_r | ATGGATCCGGGCGGGGGAGAATC |
|  | -1481_r | TAGGATCCAATGATCAACATTATTAATTTATC |
|  |  |  |
| **Reverse-transcript PCR** | P1_f | TATCTCGAGGTGTTATCTTCGCTTC |
|  | P2_f | TATCTCGAGCCCAAACAACAGTCGATTG |
|  | P3_f | TATCTCGAGGACTGTGACCTCTTGGGC |
|  | P_r | GATGGACAGTGTGGCAATGTCTC |
|  | P_r nest | TTCTTTGCTTTCGACTGCCCC |
|  |  |  |
| **Quantitative PCR** | 18S_f | CGGAGGTTCGAAGACGATCA |
|  | 18S_r | TCGCTAGTTGGCATCGTTTATG |
|  | β-actin_f | CGAGCAGGAGATGGGAACC |
|  | β-actin_f | CAACGGAAACGCTCATTGC |
|  | total_f | TCTTTCTGGACAGCTGAGGAGGTTG |
|  | total_r | CATAGAAGCAGCGGGCTTCAGTC |
|  | v1&2_f | AGCTGGACCGTAGACCTGCTAG |
|  | v1&2_r | AGCTCAAACTGGGTGGCGT |
|  | v3a_f | TCATACGGAGGATTTGATGAAGCTT |
|  | v3a_r | CTTTGCTTTCGACTGCCCCTC |
|  | v3b_f | CCTCTTGGGCGTTTTTATTGCTT |
|  | v3b_r | CTTTGCTTTCGACTGCCCCTC |
|  | v3c_f | CATACGGAGGATTTGATGAAGCCA |
|  | v3c_r | TGGGAAAATGACAAAGCGATGG |
|  | v3d_f | ATACGGAGGATTTGATGAAGGGG |
|  | v3d_r | TTTGGACAGATCAACCTCCTCAG |
|  |  |  |
| **Promoter mutations** | mE2F | CATGTTTGAATTTCCTCATAGGTGCAGCTCT |
|  | mCCAATⅠ | GAGAAATCTGACTAGTTCATCTGATGAACG |
|  | mCCAATⅡ | GCTCTTTCCCCACTAGCTGAGAATAAGCGC |
|  |  |  |
| **Ectopic expression** | R1_f | TATTCTAGAAAGCTTATGCACGTGATCAAGA |
|  | R1_r | TATTGATCAGTCGACTTATGATCCGCACATCA |
|  | R2_f | ATGAATTCCCAAGCTTATGTCGTCCACTCGCT |
|  | △29R2_f | ATGAATTCCCAAGCTTATGAAGCCACCCAGTT |
|  | △52R2_f | ATGAATTCCCTCTAGAAAGCTTATGAAGGGGCAGTCGA |
|  | R2_r | TAGGATCCCTCGAGTTAAAAATCAGCATCC |
