# Supplementary material for: Identification and Characterization of Alternative Promoters, Transcripts and Protein Isoforms of Zebrafish R2 Gene
Source: PLoS One. 2011 Aug 24;6(8):e24089. doi: 10.1371/journal.pone.0024089 (PMC3161108; doi:10.1371/journal.pone.0024089)
Supplement: Table S2 — Accession numbers of sequences used in this study. (DOC) [file pone.0024089.s005.doc]

**Table S2. Accession numbers of sequences used in this study**

| **Gene** | [**Scientific Name**](app:ds:scientific name) | **Common Name** | **Accession Number** |
| --- | --- | --- | --- |
| R2 | *Homo sapiens* | Human | NP_001025 |
| R2 | *Mus musculus* | Mouse | NP_033130 |
| R2 | *Ornithorhynchus anatinus* | [Platypus](http://www.zazzle.com/platypus_mammal_o_anatinus_tshirt-235604400796525749) | ENSOANP00000008750 |
| R2 | *Anolis carolinensis* | Green Anole | ENSACAP00000000118 |
| R2 | [*Gallus gallus*](http://species.wikimedia.org/wiki/Gallus_gallus) | Chicken | XP_419948 |
| R2 | *Xenopus tropicalis* | Clawed frog | AAH80161 |
| R2 | *Danio rerio* | Zebrafish | NP_571525 |
| R2 | [*Takifugu rubripes*](http://www.ncbi.nlm.nih.gov/Taxonomy/Browser/wwwtax.cgi?mode=Info&id=31033) | Putterfish | ENSTRUP00000020725 |
| R2 | *Tetraodon nigroviridis* | Green putterfish | ENSTNIP00000005063 |
| R2 | *Oryzias latipes* | Medaka | NP_001133495 |
| p53R2 | *Homo sapiens* | Human | NP_056528 |
| p53R2 | *Mus musculus* | Mouse | NP_955770 |
| p53R2 | *Ornithorhynchus anatinus* | [Platypus](http://www.zazzle.com/platypus_mammal_o_anatinus_tshirt-235604400796525749) | XP_001508783 |
| p53R2 | *Anolis carolinensis* | Green Anole | ENSACAP00000008780 |
| p53R2 | [*Gallus gallus*](http://species.wikimedia.org/wiki/Gallus_gallus) | domestic fowl | XP_418364 |
| p53R2 | *Xenopus tropicalis* | Clawed frog | NP_001119973 |
| p53R2 | *Danio rerio* | Zebrafish | NP_001007164 |
| p53R2 | [*Takifugu rubripes*](http://www.ncbi.nlm.nih.gov/Taxonomy/Browser/wwwtax.cgi?mode=Info&id=31033) | Putterfish | ENSTRUP00000037887 |
| p53R2 | *Tetraodon nigroviridis* | Green putterfish | CAF96041 |
| p53R2 | *Oryzias latipes* | Medaka | ENSORLP00000016486 |
| R2 | *Spisula solidissima* | Atlantic [Surf clam](http://www.nefsc.noaa.gov/publications/tm/tm142/tm142.pdf) | P07201 |
| R2 | [*Urechis caupo*](http://people.biology.ufl.edu/djulian/urechis.html) | Echiura | AAA74020 |
| R2 | *Strongylocentrotus purpuratus* | Urchin | XP_780110 |
| R2 | *Saccoglossus kowalevskii* | Acorn worm | XP_002730368 |
| R2 | [*Aedes albopictus*](http://en.wikipedia.org/wiki/Aedes_albopictus) | Asian tiger mosquito | AAD30422 |
| R2 | *Drosophila melanogaster* | Fruit fly | NP_525111 |
| R2 | *Caenorhabditis elegans* | Roundworm | P42170 |
| R2 | *Trichinella spiralis* | Trichina worm | EFV55552 |
| R2 | *Schistosoma mansoni* | blood fluke | XP_002576867 |
| R2 | *Trypanosoma brucei* | African Sleeping  Sickness rypanosome | Y10768 |
| R2 | [*Dictyostelium discoideum*](http://www.answers.com/topic/dictyostelium-discoideum) | Slime mold | AAB72227 |
| R2 | *Leishmania amazonensis* |  | AAC08302 |
| R2 | *Plasmodium falciparum* |  | P50650 |
|  | Vaccinia virus |  | P29883 |
| R2 | [*Nicotiana tabacum*](http://en.wikipedia.org/wiki/Nicotiana_tabacum) | Tobacco | P49730 |
| R2A | *Arabidopsis thaliana* | Thale cress | NP_189000 |
| R2B | *Arabidopsis thaliana* | Thale cress | AY178109 |
| TSO2 | *Arabidopsis thaliana* | Thale cress | NP_189342 |
| RNRS1 | [*Oryza sativa*](http://www.uniprot.org/taxonomy/4530) | Rice | ACC95435 |
| RNRS2 | [*Oryza sativa*](http://www.uniprot.org/taxonomy/4530) | Rice | ACC95437 |
| R2 | *Schizosaccharomyces pombe* | Fission yeast | CAA46231 |
| R2 | [*Saccharomyces cerevisiae*](http://en.wikipedia.org/wiki/Saccharomyces_cerevisiae) | Budding yeast | CAA89317 |
| R2 | [*Escherichia coli*](http://wiki.answers.com/Q/What_is_the_common_name_for_Escherichia_coli) |  | P00453 |
| Δ29R2 | *Danio rerio* | Zebrafish | JF794549 |
| Δ53R2 | *Danio rerio* | Zebrafish | JF794550 |
| R2 | *Homo sapiens* | Human | NC_000002 |
| R2 | [*Gallus gallus*](http://species.wikimedia.org/wiki/Gallus_gallus) | Chicken | NC_006090 |
| R2 | *Xenopus tropicalis* | Clawed frog | NW_003163891 |
| R2 | *Danio rerio* | Zebrafish | BX248136 |
